# Ice Cream Rounds: The implementation of peer support debriefing sessions at a Canadian medical school

**DOI:** 10.36834/cmej.69253

**Published:** 2020-08-06

**Authors:** Rashi Hiranandani, Samantha Calder-Sprackman

**Affiliations:** 1 Faculty of Medicine, University of Ottawa, Ontario, Canada; 2 Department of Emergency Medicine, University of Ottawa, Ontario, Canada; 3 Department of Family Medicine, Queen’s University, Ontario, Canada

## Implication Statement

Wellness programs exist for medical students but the opportunity to debrief challenging experiences is lacking. We piloted Ice Cream Rounds (ICRs) for University of Ottawa clerkship students during the 2018-2019 academic year to provide students a safe environment to discuss challenges. Students reported a decrease in stress, anxiety and burnout, and an improvement in collegiality as a result of ICRs. ICRs could benefit medical students at other universities. To successfully implement ICRs at your institution, we recommend obtaining funding for ice cream, having peer facilitators, and creating a safe and confidential environment where students feel comfortable to debrief challenging experiences.

## Déclaration des répercussions

Certains programmes de bien-être existent pour les étudiants en médecine, mais rares sont les occasions pour eux de raconter leurs expériences difficiles. Nous avons mené le projet pilote *Tournées de Crème Glacée* (TCG) auprès des externes de l’Université d’Ottawa durant l’année universitaire de 2018-2019. Cette initiative avait pour but de leur offrir un environnement sûr pour discuter de leurs défis. Les étudiants ont signalé une réduction de leur stress, anxiété et épuisement et une amélioration de la collégialité à la suite des TCG. Les étudiants d’autres universités pourraient sûrement bénéficier des TCG. Pour implanter avec succès des TCG dans votre établissement, nous recommandons d’obtenir des fonds pour payer la crème glacée, de recruter des facilitateurs parmi les pairs et de créer un environnement sûr et confidentiel dans lequel les étudiants se sentiront à l’aise de parler de leurs expériences difficiles.

Clerkship presents medical students with new challenges such as sleep deprivation, dealing with patient morbidity and mortality, and rotating through unfamiliar clinical environments with limited orientation. With exposure to such challenges, it is unsurprising that clerkship students have high rates of depression, burnout, anxiety, compassion fatigue, and stress.^1^^-^^3^ Although, wellness programs exist, medical students might lack opportunities to openly and consistently debrief about challenging experiences.^4^ To address this perceived wellness gap, we sought to implement peer support debriefing sessions as Ice Cream Rounds (ICRs) for clerkship students at the University of Ottawa (UOttawa).

Following research ethics exemption, we conducted an anonymous needs assessment of UOttawa clerkship students in 2018. One hundred students (32%) responded to this survey (64 respondents in third year and 36 respondents in fourth year). Respondents revealed high levels of stress: 51% stated they are very often overwhelmed or stressed in medical school, and 49% reported experiencing a challenging case in clerkship. Survey responses also confirmed that students feel a need to debrief after challenges: 66% stated that debriefing after difficult events was very important and 89% stated that medical students were their primary supports for debriefing. Overall, 71% stated they would be interested in peer support debriefing sessions, confirming the perceived wellness gap around debriefing for clerkship medical trainees.

We conducted three ICRs over the 2018-2019 academic year. All clerkship students were invited to attend. UOttawa’s medical student society provided funding for ice cream. The same staff physician and two-fourth year medical students facilitated each of the large-group sessions. The staff physician had received training from a psychiatrist to run ICRs for Emergency Medicine residents. Between five and fifteen clerkship students attended each session. Attendance and participation were voluntary. Few medical students attended all three ICRs, and the number of attendees increased as the year progressed.

**Figure 1:**
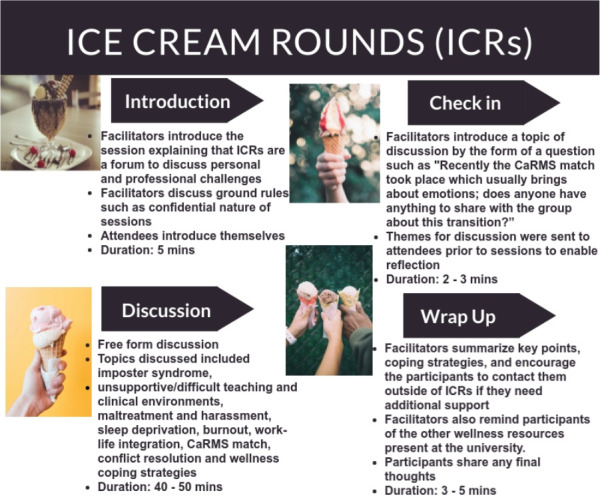
Description of Ice Cream Rounds held for UOttawa clerkship medical students

The format of the sessions was similar to those conducted for UOttawa Emergency Medicine residents^5^^,^^6^ (Figure 1). Facilitators chose topics based on what students were experiencing. The check-in was brief to introduce the conversation. Despite having pre-set themes for the rounds, students often guided topics of conversation based on the current challenges they were experiencing. After each session, we distributed a questionnaire to evaluate the usefulness of ICRs. Eighteen (78%) students responded to this survey. All respondents were glad ICRs existed and would recommend attendance to other medical students. Of the 18 students, 94% reported ICRs greatly benefitted them, stating that ICRs normalized challenges in medical training; 55% stated ICRs greatly decreased their stress; 72% stated ICRs greatly reduced their feelings of burnout and anxiety in medical school; and 94% expressed that ICRs greatly improved collegiality between students.

Attendance to ICRs was lower than expected, which may be attributed to student time constraints or potential discomfort disclosing challenges with peers in this type of setting. Increasing advertising efforts and obtaining faculty approved protected time for ICRs might improve attendance in the future. ICRs can be adapted for other institutions to address the wellness gap around debriefing during medical training.
